# Clinical utility of exome sequencing in hearing loss: a retrospective cohort study

**DOI:** 10.3389/fgene.2025.1643537

**Published:** 2025-09-10

**Authors:** Chang Liu, Yanlin Huang, Anpeng Fu, Yunan Wang, Jing Wu, Yan Zhang, Li Du, Hongke Ding, Lihua Yu, Fake Li, Yiming Qi, Yuan Liu, Xingwang Wang, Yukun Zeng, Ling Liu, Ying Xiong, Yuanling Liu, Xin Zhao, Liyuan Fang, Jiayi Jian, Aihua Yin, Yanqin You

**Affiliations:** ^1^ Medical Genetic Center, Guangdong Women and Children Hospital, Guangzhou, Guangdong, China; ^2^ Guangzhou Key Laboratory of Prenatal Screening and Diagnosis, Guangdong Women and Children Hospital, Guangzhou, Guangdong, China; ^3^ Prenatal Diagnosis Center, Guangdong Women and Children Hospital, Guangzhou, Guangdong, China; ^4^ Department of Obstetrics and Gynecology, First Medical Center of Chinese PLA General Hospital, Beijing, China

**Keywords:** hearing loss, exome sequencing, molecular analysis, clinical evaluation, reanalysis

## Abstract

**Background:**

Hearing loss (HL) is a prevalent sensorineural disorder with a highly heterogeneous etiology. Next-generation sequencing (NGS) has revolutionized the genetic testing landscape for diseases characterized by high genetic and allelic heterogeneity, enabling the simultaneous screening of hundreds of genes.

**Methods:**

One hundred and seventy-one unrelated patients with non-syndromic or syndromic HL were enrolled in this study. Exome sequencing (ES) was applied to explore molecular etiology in the cohort, and clinical reports were provided by geneticists and genetic counselors. Multidisciplinary team forums were conducted to ensure accurate diagnoses and improved patient management.

**Results:**

The molecular cause of HL was determined in 78 of 171 probands (45.6%): 54 with an autosomal recessive (AR) inheritance pattern, 23 with an autosomal dominant (AD) pattern, and 1 with both AR/AD inheritance patterns. Candidate variants in 33 genes were identified in the study cohort: 14 with an AR inheritance pattern, 18 with an AD pattern, and 1 with both AR/AD inheritance patterns. Twenty-eight of the variants identified in the study were novel.

**Conclusion:**

Exome sequencing facilitates genetic diagnosis and improves the management of patients with HL in clinical practice. Identifying the etiology of HL may improve patient care, refine genetic counseling, and facilitate the estimation of recurrence risk.

## 1 Introduction

According to estimates from the World Health Organization, approximately 466 million individuals globally are affected by disabling hearing loss (HL), including 34 million children under the age of fifteen years (Wilson et al., 2017). HL is a prevalent sensorineural disorder with implications that extend far beyond sensory impairment (World Health Organization, 2018). Insufficient auditory stimulation or inadequate language exposure during early childhood may alter brain connectivity and processing; thus, children who have not acquired adequate auditory stimulation may encounter significant challenges in their subsequent linguistic acquisition, cognitive development, and psychosocial functioning (World Health Organization, 2018; Kral and O’Donoghue, 2010; Lieu et al., 2020). HL can manifest at any stage of life, impairing communication, affecting social interactions, and creating difficulties in professional settings (World Health Organization, 2018).

The complex etiology of HL, coupled with the highly variable and often overlapping presentations of different types of HL, poses challenges for traditional clinical diagnosis (Alford et al., 2014). It is estimated that up to 60% of educationally significant congenital and early-onset HL is associated with genetic factors (Alford et al., 2014; Schimmenti et al., 2004). Although the role of genetic factors in adult-onset HL remains less clear, an increasing number of susceptibility loci have been identified, and a substantial proportion of cases have been attributed to genetic causes (Alford et al., 2014; Michels et al., 2019). Approximately 120 non-syndromic loci have been identified in humans (hereditaryhearingloss.org), and over 400 genetic syndromes include HL as a characteristic feature (Alford et al., 2014; García-García et al., 2020). Recent recommendations advocate genetic testing as the initial diagnostic step for children with bilateral sensorineural hearing loss (SNHL), following cytomegalovirus (CMV) testing. Identifying the genetic etiology of HL can provide several potential benefits for patients and their families, including improved clinical management, informed planning for future medical and educational needs, and more accurate estimation of recurrence risk (Alford et al., 2014). Furthermore, given the rapid advancement of SNHL gene therapy research, understanding the genetic etiology of HL is crucial for assessing the eligibility for imminent clinical trials.

Traditional molecular diagnostic tests for HL primarily involved genotyping or DNA sequencing to identify specific HL variants or screen a limited number of genes associated with it. In the last decade, next-generation sequencing (NGS) has revolutionized the genetic testing landscape for diseases characterized by high genetic and allelic heterogeneity, such as HL, enabling the simultaneous screening of hundreds of genes (García-García et al., 2020). In the present study, exome sequencing (ES) was utilized to facilitate genetic diagnosis and improve the management of patients with HL in clinical practice.

## 2 Materials and methods

### 2.1 Ethics statement

The study was approved by the Medical Ethics Committee of Guangdong Women and Children Hospital. Written informed consent was obtained from all participants and their parents or legal guardians (in the case of children under 18). The authors had access to identifiable patient information, which was anonymized prior to submission. All the procedures conducted in the study adhered to the Declaration of Helsinki, as previously described (Liu et al., 2022).

### 2.2 Patients and samples

A total of 171 unrelated patients diagnosed with non-syndromic or syndromic HL were enrolled from January 2017 to September 2024 under an institutional review board-approved protocol of informed consent. A comprehensive history and physical examination were obtained for each participant, including clinical history, severity of HL, age and cause of onset, infection history, exposure to aminoglycoside antibiotics, and genetic factors associated with hearing impairment (Yin et al., 2014). The recruited patients exhibited sensorineural or mixed HL. The severity of HL was established as mild (hearing thresholds 26 dB–40 dB), moderate (hearing thresholds 41 dB–60 dB), severe (hearing thresholds 61 dB–80 dB), or profound (hearing thresholds more than 80 dB). A stepwise approach was provided as an alternative (Yin et al., 2014), and four patients in the study cohort opted for this approach and were pre-screened for the *GJB2*, *SLC26A4*, and *MT-RNR1* gene variants prior to ES.

Peripheral blood samples were collected from the patients and their first-degree relatives if available. Genomic DNA was extracted using the SolPure Blood DNA Kit (Magen, Shanghai, China), following the manufacturer’s instructions, which included optional RNase treatment. The DNA was then fragmented using a Q800R Sonicator (Qsonica, CT, United States). The paired-end libraries were prepared following the Illumina library preparation protocol.

### 2.3 Exome sequencing

All cases underwent genetic evaluations using either whole-exome sequencing (WES) or clinical exome sequencing (CES). Custom-designed NimbleGen SeqCap probes (Roche NimbleGen, Madison, WI) were used for in-solution hybridization to enrich target sequences for WES or, in the case of clinical ES, the target sequences included ∼5,000 genes that were potentially associated with known Mendelian genetic diseases (AmCare Genomic Laboratory, Guangzhou, China). Low-quality reads (Phred score < Q20) were removed before demultiplexing. Sequences were aligned to the hg19 reference genome using NextGENe software (SoftGenetics, State College, PA) with standard parameters for single-nucleotide variant (SNV) and insertion/deletion (indel) detection.

### 2.4 Data analysis

The resulting sequencing data were analyzed using NextGENe software with reference to the human assembly GRCh37/hg19. This software program performs the alignment, variant calling, and annotation of the variants. The annotated variants were filtered according to a minor allele frequency (MAF) value >0.01, and the frequency of the variants was explored in the gnomAD v4.1.0 database (https://gnomad.broadinstitute.org/). To classify the variants, we considered their annotation in dbSNP (www.ncbi.nlm.nih.gov/SNP/); their description in ClinVar (https://www.ncbi.nlm.nih.gov/clinvar/), VarSome (https://Varsome.com/), and HGMD (http://www.hgmd.cf.ac.uk/); and the variant type, as previously described (García-García et al., 2020). The detailed process and quality control system for identifying the candidate variants were applied as previously described (Liu et al., 2022). Variant classification adhered to the 2015 ACMG/AMP guidelines (Richards et al., 2015; Tavtigian et al., 2020). Copy number variation (CNV) identification utilized a CNVkit (Talevich et al., 2016). AnnotSV annotated the detected CNVs in each tested sample for clinical interpretation (Geoffroy et al., 2018). Candidate CNV clinical significance was evaluated following the ACMG/ClinGen criteria (Riggs et al., 2020). Diagnostic variants were validated by Sanger sequencing or quantitative polymerase chain reaction (qPCR) when applicable.

### 2.5 Reanalysis and reclassification

The purpose of variant reinterpretation was to reassess the pathogenicity of the variants. All the variants were reinterpreted according to the standards and guidelines recommended by the ACMG/AMP. Negative and uncertain results were reanalyzed from the raw sequencing data stored as compressed fastq files (Nambot et al., 2018). All variants of the final analysis file were interpreted. The reclassification focused on variants that were previously and newly reported as pathogenic/probably pathogenic (P/LP) in updated public databases of clinical interest (OMIM, https://omim.org/; ClinVar, http://www.ncbi.nlm.nih.gov/clinvar; DECIPHER, https://www.deciphergenomics.org/; HGMD, http://www.hgmd.cf.ac.uk/) or as affecting the well-established human disease genes (Nambot et al., 2018). The interpretation was then extended to all other variants, namely, those not meeting the diagnostic interpretation criteria (Nambot et al., 2018). Variants that remained undiagnosed were subject to periodic re-interpretation.

## 3 Results

### 3.1 Cohort description

A total of 171 unrelated patients were enrolled in the study, and the characteristics of the study cohort are summarized in Table 1. One hundred and thirty-four patients were affected with non-syndromic hearing loss (NSHL), and 37 were affected with syndromic hearing loss (SHL). The degree of HL in the patients varied, and severe-to-profound HL was observed in the majority (78.4%, 134/171). In addition, pre-lingual HL was detected in 80.6% (145/171) of the patients.

**TABLE 1:** Characteristics of the study cohort.

Characteristic	No.	%
All	171	100.0
Sex		
M	94	55.0
F	77	45.0
Family history		
Yes	27	15.8
No	144	84.2
Onset		
Pre-lingual (≤3 years)	145	84.8
Post-lingual (>3 years)	26	15.2
Bilateral symmetric	124	72.5
Bilateral asymmetric	44	25.7
No record	3	1.8
Stability		
Stable	125	73.1
Fluctuating	40	23.4
No record	6	3.5
Aminoglycoside exposure		
Yes	21	12.3
No	120	70.2
Uncertain	30	17.5
Severity		
Mild	4	2.3
Moderate	32	18.7
Severe	90	52.6
Profound	44	25.7
No record	1	0.6
Rehabilitation		
Hearing aid	69	40.4
Cochlear implantation	67	39.2
Both	7	4.1
Not applied	28	16.4
Newborn hearing screening result		
Pass	30	17.5
Referral	78	45.6
Not applied/no record	63	36.8

### 3.2 Diagnostic findings

**FIGURE 1:**
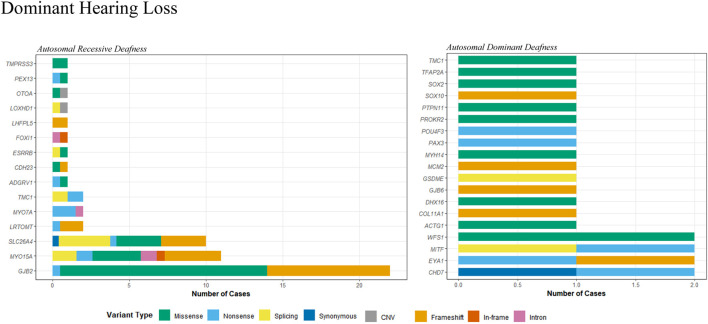
Distribution of variant types in genes associated with autosomal recessive and dominant hearing loss.

The etiologic diagnosis for HL has been identified for 78 out of 171 probands (45.6%), among which 55 had an autosomal recessive (AR) inheritance pattern and 23 had an autosomal dominant (AD) pattern. Table 2 contains the clinical data and genetic evaluations of the diagnosed patients. Candidate variants in 33 genes were identified in the study cohort, with 14 having an AR inheritance pattern and 19 having an AD pattern (Figure 1). A total of 91 candidate variants were identified, including 33 missense, 21 frameshift, 16 nonsense, 9 canonical splicing, 4 intronic, 3 CNVs, 2 in-frame, 2 synonymous, and 1 multi-nucleotide variants. Of the 91 variants, 28 were novel (Table 4).

**TABLE 2:** Clinical data and genetic evaluations of the diagnosed patients.

Patient	Sex	Age	Onset	Severity	Family history	Diagnosis	Gene	Allele 1	Allele 2
(A) Patients diagnosed with autosomal recessive deafness									
P1	M	25 y	Pre-lingual	Severe	No	Deafness, autosomal recessive 4, with enlarged vestibular aqueduct/Pendred syndrome	*SLC26A4* (NM_000441.2)	c.919–2A>G	c.589G>A (p.Gly197Arg)
P2	M	26 y	Pre-lingual	Profound	Yes	Deafness, autosomal recessive 4, with enlarged vestibular aqueduct/Pendred syndrome	*SLC26A4* (NM_000441.2)	c.919–2A>G	c.1692dup (p.Cys565MetfsTer9)
P3	M	27 y	Pre-lingual	Profound	Yes	Deafness, autosomal recessive 4, with enlarged vestibular aqueduct/Pendred syndrome	*SLC26A4* (NM_000441.2)	c.919–2A>G	c.919–2A>G
P4	F	9 y	Pre-lingual	Severe	No	Deafness, autosomal recessive 4, with enlarged vestibular aqueduct/Pendred syndrome	*SLC26A4* (NM_000441.2)	c.563T>C (p.Ile188Thr)	c.1547dup (p.Ser517PhefsTer10)
								c.1746del (p.Ala584ArgfsTer2)	
P5	M	25 y	Pre-lingual	Severe	Yes	Deafness, autosomal recessive 4, with enlarged vestibular aqueduct/Pendred syndrome	*SLC26A4* (NM_000441.2)	c.919–2A>G	c.259G>T (p.Asp87Tyr)
P6	M	8 y	Pre-lingual	Profound	No	Deafness, autosomal recessive 4, with enlarged vestibular aqueduct/Pendred syndrome	*SLC26A4* (NM_000441.2)	c.919–2A>G	c.1975G>C (p.Val659Leu)
P7	F	26 y	Pre-lingual	Profound	Yes	Deafness, autosomal recessive 4, with enlarged vestibular aqueduct/Pendred syndrome	*SLC26A4* (NM_000441.2)	c.919–2A>G	c.1229C>T (p.Thr410Met)
P8	M	5 y	Post-lingual	Severe	No	Deafness, autosomal recessive 4, with enlarged vestibular aqueduct/Pendred syndrome	*SLC26A4* (NM_000441.2)	c.589G>C (p.Gly197Arg)	c.754T>C (p.Ser252Pro)
P9	M	5 y	Pre-lingual	Profound	No	Deafness, autosomal recessive 4, with enlarged vestibular aqueduct/Pendred syndrome	*SLC26A4* (NM_000441.2)	c.414del (p.Gly139AspfsTer6)	c.600G>A (p.Gln200 =)
P10	M	27 y	Pre-lingual	Profound	No	Deafness, autosomal recessive 4, with enlarged vestibular aqueduct/Pendred syndrome	*SLC26A4* (NM_000441.2)	c.919–2A>G	c.2086C>T (p.Gln696Ter)
P11	F	2 y	Pre-lingual	Profound	Yes	Deafness, autosomal recessive 1A	*GJB2* (NM_004004.6)	c.235del (p.Leu79CysfsTer3)	c.235del (p.Leu79CysfsTer3)
P12	M	26 y	Pre-lingual	Profound	No	Deafness, autosomal recessive 1A	*GJB2* (NM_004004.6)	c.235del (p.Leu79CysfsTer3)	c.508_511dup (p.Ala171GlufsTer40)
P13	M	27 y	Pre-lingual	Profound	No	Deafness, autosomal recessive 1A	*GJB2* (NM_004004.6)	c.299_300del (p.His100ArgfsTer14)	c.299_300del (p.His100ArgfsTer14)
P14	M	24 y	Pre-lingual	Profound	No	Deafness, autosomal recessive 1A	*GJB2* (NM_004004.6)	c.109G>A (p.Val37Ile)	c.109G>A (p.Val37Ile)
P15	M	6 y	Pre-lingual	Profound	No	Deafness, autosomal recessive 1A	*GJB2* (NM_004004.6)	c.109G>A (p.Val37Ile)	c.109G>A (p.Val37Ile)
P16	M	22 y	Pre-lingual	Severe	No	Deafness, autosomal recessive 1A	*GJB2* (NM_004004.6)	c.235del (p.Leu79CysfsTer3)	c.508_511dup (p.Ala171GlufsTer40)
P17	F	10 y	Post-lingual	Moderate	No	Deafness, autosomal recessive 1A	*GJB2* (NM_004004.6)	c.109G>A (p.Val37Ile)	c.109G>A (p.Val37Ile)
						Chromosome 22q11.2 microduplication syndrome	22q11.21 (chr22:18893887-21386101)×3		
P18	M	5 y	Pre-lingual	Moderate	No	Deafness, autosomal recessive 1A	*GJB2* (NM_004004.6)	c.109G>A (p.Val37Ile)	c.109G>A (p.Val37Ile)
						Peroxisome biogenesis disorder 11B	*PEX13* (NM_002618.4)	c.676C>T (p.Arg226Ter)	c.880C>T (p.Arg294Trp)
P19	F	8 y	Pre-lingual	Moderate	No	Deafness, autosomal recessive 1A	*GJB2* (NM_004004.6)	c.109G>A (p.Val37Ile)	c.109G>A (p.Val37Ile)
P20	M	5 y	Pre-lingual	Severe	No	Deafness, autosomal recessive 1A	*GJB2* (NM_004004.6)	c.235del (p.Leu79CysfsTer3)	c.427C>T (p.Arg143Trp)
P21	M	10 y	Pre-lingual	Severe	Yes	Deafness, autosomal recessive 1A	*GJB2* (NM_004004.6)	c.176_191del (p.Gly59AlafsTer18)	c.235del (p.Leu79CysfsTer3)
P22	F	21 y	Post-lingual	Severe	No	Deafness, autosomal recessive 1A	*GJB2* (NM_004004.6)	c.109G>A (p.Val37Ile)	c.109G>A (p.Val37Ile)
P23	F	4 y	Pre-lingual	Moderate	No	Deafness, autosomal recessive 1A	*GJB2* (NM_004004.6)	c.109G>A (p.Val37Ile)	c.109G>A (p.Val37Ile)
						Deafness, autosomal recessive 22	*OTOA* (NM_144672.4)	c.119C>G (p.Ala40Gly)	16p12.2 (chr16:21415697-21747711)×1 including OTOA exon1-22
P24	F	10 y	Pre-lingual	Moderate	No	Deafness, autosomal recessive 1A	*GJB2* (NM_004004.6)	c.109G>A (p.Val37Ile)	c.109G>A (p.Val37Ile)
P25	F	48 y	Pre-lingual	Severe	No	Deafness, autosomal recessive 1A	*GJB2* (NM_004004.6)	c.109G>A (p.Val37Ile)	c.379C>T (p.Arg127Cys)
P26	F	24 y	Pre-lingual	Profound	No	Deafness, autosomal recessive 1A	*GJB2* (NM_004004.6)	c.235del (p.Leu79CysfsTer3)	c.235del (p.Leu79CysfsTer3)
P27	M	42 y	Post-lingual	Moderate	No	Deafness, autosomal recessive 1A	*GJB2* (NM_004004.6)	c.109G>A (p.Val37Ile)	c.109G>A (p.Val37Ile)
P28	M	6 y	Pre-lingual	Moderate	No	Deafness, autosomal recessive 1A	*GJB2* (NM_004004.6)	c.571T>C (p.Phe191Leu)	c.109G>A (p.Val37Ile)
P29	M	8 y	Pre-lingual	Severe	No	Deafness, autosomal recessive 1A	*GJB2* (NM_004004.6)	c.235del (p.Leu79CysfsTer3)	c.235del (p.Leu79CysfsTer3)
P30	F	8 y	Pre-lingual	Moderate	No	Deafness, autosomal recessive 1A	*GJB2* (NM_004004.6)	c.109G>A (p.Val37Ile)	c.109G>A (p.Val37Ile)
P31	F	27 y	Pre-lingual	Severe	Yes	Deafness, autosomal recessive 1A	*GJB2* (NM_004004.6)	c.235del (p.Leu79CysfsTer3)	c.139G>T (p.Glu47Ter)
P32	F	1 y	Pre-lingual	Moderate	No	Deafness, autosomal recessive 1A	*GJB2* (NM_004004.6)	c.109G>A (p.Val37Ile)	c.109G>A (p.Val37Ile)
P33	F	26 y	Pre-lingual	Profound	Yes	Deafness, autosomal recessive 3	*MYO15A* (NM_016239.4)	c.3524dup (p.Ser1176ValfsTer14)	c.3761_3764dup (p.Gly1256HisfsTer60)
P34	F	5 y	Pre-lingual	Severe	No	Deafness, autosomal recessive 3	*MYO15A* (NM_016239.4)	c.10250_10252del (p.Ser3417del)	c.8138T>G (p.Leu2713Arg)
P35	M	25 y	Pre-lingual	Profound	No	Deafness, autosomal recessive 3	*MYO15A* (NM_016239.4)	c.3524dup (p.Ser1176ValfsTer14)	c.3524dup (p.Ser1176ValfsTer14)
P36	F	6 y	Post-lingual	Moderate	No	Deafness, autosomal recessive 3	*MYO15A* (NM_016239.4)	c.1203C>A (p.Tyr401Ter)	c.5649 + 1G>T
P37	M	6 y	Pre-lingual	Profound	No	Deafness, autosomal recessive 3	*MYO15A* (NM_016239.4)	c.9572G>A (p.Arg3191His)	c.9156 + 91A>G
P38	F	11 y	Pre-lingual	Severe	No	Deafness, autosomal recessive 3	*MYO15A* (NM_016239.4)	c.3524dup (p.Ser1176ValfsTer14)	c.3866 + 5G>A
P39	M	24 y	Pre-lingual	Profound	No	Deafness, autosomal recessive 3	*MYO15A* (NM_016239.4)	c.7744C>T (p.Gln2582Ter)	c.8971C>G (p.Pro2991Ala)
						Deafness, autosomal dominant 70	*MCM2* (NM_004526.4)	c.993dup (p.Cys332ValfsTer23)	
P40	M	9 y	Pre-lingual	Severe	No	Deafness, autosomal recessive 3	*MYO15A* (NM_016239.4)	c.5275A>C (p.Ser1759Arg)	c.10250_10252del (p.Ser3417del)
P41	F	34 y	Pre-lingual	Profound	No	Deafness, autosomal recessive 3	*MYO15A* (NM_016239.4)	c.3524dup (p.Ser1176ValfsTer14)	c.3524dup (p.Ser1176ValfsTer14)
P42	F	26 y	Pre-lingual	Profound	No	Deafness, autosomal recessive 3	*MYO15A* (NM_016239.4)	c.6177 + 1G>T	c.6292G>A (p.Asp2098Asn)
P43	M	3 y	Pre-lingual	Severe	No	Deafness, autosomal recessive 22	*MYO7A* (NM_000260.4)	c.4398G>A (p.Trp1466Ter)	c.4485G>A (p.Trp1495Ter)
P44	F	35 y	Post-lingual	Moderate	No	Deafness, autosomal recessive 22	*MYO7A* (NM_000260.4)	c.291T>G (p.Tyr97Ter)	c.285 + 5G>C
P45	M	1 y	Pre-lingual	Severe	No	Deafness, autosomal recessive 63	*LRTOMT* (NM_001145308.5)	c.566del (p.Ile189ThrfsTer8)	c.566del (p.Ile189ThrfsTer8)
P46	M	4 y	Pre-lingual	Profound	No	Deafness, autosomal recessive 63	*LRTOMT* (NM_001145308.5)	c.566del (p.Ile189ThrfsTer8)	c.655C>T (p.Arg219Ter)
P47	M	43 y	Pre-lingual	Profound	No	Deafness, autosomal recessive 7	*TMC1* (NM_138691.3)	c.453 + 2T>C	c.453 + 2T>C
P48	M	4 y	Pre-lingual	Profound	No	Deafness, autosomal recessive 7	*TMC1* (NM_138691.3)	c.1547G>A (p.Trp516Ter)	c.1547G>A (p.Trp516Ter)
P49	F	21 y	Post-lingual	Moderate	Yes	Usher syndrome, type 2C	*ADGRV1* (NM_032119.4)	c.18374A>C (p.His6125Pro)	c.10119T>G (p.Tyr3373Ter)
						Deafness, autosomal recessive 3	*MYO15A* (NM_016239.4)	c.9690 + 1G>A	c.9590G>T (p.Ser3197Ile)
P50	F	3 y	Pre-lingual	Severe	No	Usher syndrome, type 1D	*CDH23* (NM_022124.6)	c.1606C>T (p.Arg536Trp)	c.8371del (p.Leu2791TrpfsTer46)
P51	M	9 y	Pre-lingual	Severe	Yes	Deafness, autosomal recessive 8/10	*TMPRSS3* (NM_001256317.3)	c.346G>A (p.Val116Met)	c.1075G>A (p.Ala359Thr)
P52	M	7 y	Pre-lingual	Moderate	No	Deafness, autosomal recessive 77	*LOXHD1* (NM_001384474.1)	c.611–2A>T	Exon 38 deletion
P53	F	23 y	Pre-lingual	Profound	No	Deafness, autosomal recessive 35	*ESRRB* (NM_001379180.1)	c.578-1G>C	c.809G>A (p.Arg270Gln)
P54	F	6 y	Pre-lingual	Severe	No	Deafness, autosomal recessive 4, with enlarged vestibular aqueduct	*FOXI1* (NM_012188.5)	c.575–522C>G	c.483_485del (p.Asn161del)
P55	F	2 y	Pre-lingual	Profound	No	Deafness, autosomal recessive 67	*LHFPL5* (NM_182548.4)	c.619del (p.Leu207SerfsTer37)	c.619del (p.Leu207SerfsTer37)

Patient	Sex	Age	Onset	Severity	Family history	Diagnosis	Gene	Allele 1
Patients diagnosed with autosomal dominant deafness								
P56	F	28 y	Pre-lingual	Severe	No	Deafness, autosomal dominant 20/26	*ACTG1* (NM_001614.5)	c.548G>A (p.Arg183Gln)
P57	M	3 y	Pre-lingual	Moderate	Yes	Deafness, autosomal dominant 6/14/38	*WFS1* (NM_006005.3)	c.2590G>A (p.Glu864Lys)
P58	F	29 y	Post-lingual	Moderate	Yes	Deafness, autosomal dominant 6/14/38	*WFS1* (NM_006005.3)	c.2389G>A (p.Asp797Asn)
P59	F	9 y	Pre-lingual	Severe	No	Waardenburg syndrome, type 2A	*MITF* (NM_001354604.2)	c.1031 + 1G>A
P60	F	13 y	Post-lingual	Moderate	Yes	Waardenburg syndrome, type 2A	*MITF* (NM_001354604.2)	c.1084C>T (p.Arg362Ter)
P61	M	56 y	Post-lingual	Severe	Yes	Deafness, autosomal dominant 36	*TMC1* (NM_138691.3)	c.1714G>A (p.Asp572Asn)
P62	F	8 y	Pre-lingual	Profound	No	Deafness, autosomal dominant 3B	*GJB6* (NM_001110219.3)	c.228del (p.Trp77GlyfsTer5)
P63	M	2 y	Pre-lingual	Profound	No	Neuromuscular disease and ocular or auditory anomalies with or without seizures	*DHX16* (NM_003587.5)	c.2474C>T (p.Ser825Phe)
P64	F	6 y	Pre-lingual	Profound	No	Hypogonadotropic hypogonadism 5 with or without anosmia	*CHD7* (NM_017780.4)	c.7879C>T (p.Arg2627Ter)
P65	F	5 y	Pre-lingual	Severe	No	CHARGE syndrome	*CHD7* (NM_017780.4)	c.4353G>C (p.Gly1451 =)
P66	F	31 y	Post-lingual	Mild	No	Osteogenesis imperfecta, type I/III	*COL1A1* (NM_000088.4)	c.1128del (p.Gly377AlafsTer164)
P67	F	2 y	Pre-lingual	Moderate	No	LEOPARD syndrome	*PTPN11* (NM_002834.5)	c.1403C>T (p.Thr468Met)
P68	M	9 y	Post-lingual	Moderate	No	Branchio-oculo-facial syndrome	*TFAP2A* (NM_001372066.1)	c.716G>A (p.Arg239Gln)
P69	M	25 y	Pre-lingual	Profound	Yes	Branchio-otic syndrome 1	*EYA1* (NM_000503.6)	c.922C>T (p.Arg308Ter)
P70	M	5 y	Pre-lingual	Severe	No	Branchio-otic syndrome 1	*EYA1* (NM_000503.6)	c.473del (p.Pro158LeufsTer83)
P71	F	2 y	Pre-lingual	Moderate	No	Microphthalmia, syndromic 3/optic nerve hypoplasia and abnormalities of the central nervous system	*SOX2* (NM_003106.4)	c.137A>G (p.Asn46Ser)
P72	M	7 m	Pre-lingual	Severe	No	Waardenburg syndrome	*SOX10* (NM_006941.4)	c.549_567del (p.Gln185ProfsTer95)
P73	F	7 y	Pre-lingual	Severe	No	Waardenburg syndrome	*SOX10* (NM_006941.4)	c.580del (p.Glu194ArgfsTer92)
P74	M	25 y	Post-lingual	Severe	Yes	Deafness, autosomal dominant 4A	*MYH14* (NM_001077186.2)	c.289_290insTGG (p.Gln97delinsLeuGlu)
P75	M	29 y	Post-lingual	Moderate	Yes	Deafness, autosomal dominant 15	*POU4F3* (NM_002700.3)	c.952del (p.Val318Ter)
P76	M	8 y	Pre-lingual	Severe	No	Hypogonadotropic hypogonadism 3 with or without anosmia	*PROKR2* (NM_144773.4)	c.533G>C (p.Trp178Ser)
P77	F	1 y	Pre-lingual	Moderate	Yes	Waardenburg syndrome, type 1	*PAX3* (NM_181458.4)	c.784C>T (p.Arg262Ter)
P78	F	44 y	Post-lingual	Severe	Yes	Deafness, autosomal dominant 5	*GSDME* (NM_001127453.2)	c.991–2A>G

Fifty-four probands carried bi-allelic pathogenic or likely pathogenic variants associated with an AR pattern of inheritance (Table 2A). Fifty-one cases presented with NSHL. These were linked to *GJB2* (22 cases), *SLC26A4* (10 cases), *MYO15A* (10 cases), *MYO7A* (2 cases), *LRTOMT* (2 cases), *TMC1* (2 cases), and 1 case in each of the *ADGRV1*, *CDH23*, *TMPRSS3*, *LOXHD1*, *ESRRB*, *FOXI1*, *PEX13*, *OTOA*, and *LHFPL5* genes (Figure 1). Of these, three cases may have had more than one gene that contributed to the HL. In patient 18, a homozygous c.109G>A (p.Val37Ile) variant in *GJB2* was identified, in addition to two P/LP variants in the *PEX13* gene. Similarly, a homozygous c.109G>A (p.Val37Ile) variant in *GJB2* was identified in patient 23, in addition to two LP/VUS variants in the *OTOA* gene. Another patient (P49) with Usher syndrome was found to carry LP variants in the *ADGRV1* gene, in addition to two LP/VUS variants in the *MYO15A* gene.

Twenty-three patients were identified with autosomal dominant HL (Table 2B). Eight cases presented with NSHL, and 15 patients presented with SHL. They were linked to *CHD7* (two cases), *EYA1* (two cases), *MITF* (two cases), *WFS1* (two cases), and one case in each of the *ACTG1*, *TMC1*, *GJB6*, *DHX16*, *COL1A1*, *PTPN11*, *TFAP2A*, *SOX2*, *SOX10*, *MYH14*, *POU4F3*, *PROKR2*, *PAX3*, and *GSDME* genes (Figure 1). Moreover, one patient (P39) may have had HL contributions from both a gene associated with AD inheritance and a gene associated with AR inheritance. A VUS variant, c.993dup, in the *MCM2* gene was identified, in addition to two LP/VUS variants in the *MYO15A* gene.

Identifying the genetic causes of HL enabled the recommendation of personalized treatment options, such as cochlear implants and hearing aids. Of the 78 cases with positive genetic diagnoses, 73 patients (93.6%) adopted rehabilitation approaches and achieved favorable therapeutic outcomes. Moreover, after genetic counseling, 27 couples utilized preimplantation genetic diagnosis or prenatal diagnosis after identifying the pathogenic variants, enabling informed reproductive choices.

### 3.3 Undiagnosed probands by ES

Reanalysis was performed for patients with a non-positive result, and the reclassification was carried out for all variants in cases with positive or uncertain results to reassess the pathogenicity of the variants. A total of 93 probands still remained undiagnosed after follow-up studies (Table 3), with 9.7% (9/93) showing inconclusive findings due to limited variant pathogenic evidence. For instance, patient 91 harbored a loss-of-function variant, c.2953delG (p.Glu985SerfsTer6), in the *MACF1* gene with an AD inheritance pattern. The variant was not found in gnomAD exomes/genomes and was predicted as damaging by multiple computational algorithms in VarSome, but further studies are needed to determine its pathogenicity. A similar situation was observed for patients 81, 88, and 95. Patient 90 harbored a homozygous c.6070G>A (p.Ala2024Thr) variant in the *PTPRQ* gene. The variant was not found in gnomAD exomes/genomes, but further studies are needed to determine its pathogenicity. Patient 92 carried two VUS variants in the *DIAPH3* gene with an AD inheritance pattern. The missense variant c.1124C>T (p.Pro375Leu) was absent from gnomAD exomes/genomes and was predicted to be deleterious by multiple computational algorithms in VarSome. The c.180 + 5G>T variant was also absent in gnomAD exomes/genomes and scored 0.867 by delta score of donor loss in spliceAI. It was predicted to affect mRNA transcription and splicing, but additional studies are needed to determine its pathogenicity.

**TABLE 3:** Undiagnosed probands by exome sequencing.

Patient	Sex	Age	Onset	Severity	Family history	Inheritance pattern	Gene-associated diseases	Gene	Allele 1	Classification	Criteria
P79	F	3 y	Pre-lingual	Severe	Yes	AR	Deafness, autosomal recessive 8/10	*TMPRSS3* (NM_001256317.3)	c.316C>T (p.Arg106Cys)	LP	PM3_S + PP1+PP3
P80	M	3 y	Pre-lingual	Profound	No	AR	Deafness, autosomal recessive 12/Usher syndrome, type 1D	*CDH23* (NM_022124.6)	c.7661-1G>C	LP	PVS1+PM2_P
						AR	Deafness, autosomal recessive 1A	*GJB2* (NM_004004.6)	c.109G>A (p.Val37Ile)	P	PS4+PM3+PP1_S + PP3
P81	M	35 y	Pre-lingual	Severe	No	AD; AR	Tietz albinism-deafness syndrome	*MITF* (NM_001354604.2)	c.1072G>A (p.Val358Met)	VUS	PM2_P + PP3
P82	M	29 y	Pre-lingual	Severe	No	AR	Deafness, autosomal recessive 23/Usher syndrome, type 1F	*PCDH15* (NM_001384140.1)	c.5041_5044dup (p.Asn1682ArgfsTer17)	VUS	PVS1_M + PM2_P
						AR	Deafness, autosomal recessive 1A	*GJB2* (NM_004004.6)	c.109G>A (p.Val37Ile)	P	PS4+PM3+PP1_S + PP3
P83	M	3 y	Pre-lingual	Severe	No	AD	Deafness, autosomal dominant 76	*PLS1* (NM_001145319.2)	c.1775G>A (p.Arg592Gln)	VUS	PM2_P + PP3_M
						AR	Deafness, autosomal recessive 1A	*GJB2* (NM_004004.6)	c.109G>A (p.Val37Ile)	P	PS4+PM3+PP1_S + PP3
P84	F	22 y	Post-lingual	Severe	No	AD	Deafness, autosomal dominant 74	*PDE1C* (NM_001191057.4)	c.1174A>C (p.Met392Leu)	VUS	PM2_P
						AR	Deafness, autosomal recessive 1A	*GJB2* (NM_004004.6)	c.109G>A (p.Val37Ile)	P	PS4+PM3+PP1_S + PP3
P85	M	4 y	Pre-lingual	Profound	No	AR	Cone-rod dystrophy and hearing loss	*CEP78* (NM_001330691.3)	c.1251 + 5G>A	P	PVS1(RNA)+PM2_P + PM3+PP4
						AR	Deafness, autosomal recessive 1A	*GJB2* (NM_004004.6)	c.109G>A (p.Val37Ile)	P	PS4+PM3+PP1_S + PP3
P86	F	4 y	Pre-lingual	Severe	No	AD; AR	Deafness, autosomal dominant 8/12; Deafness, autosomal recessive 21	*TECTA* (NM_005422.4)	c.3605C>T (p.Ser1202Phe)	VUS	PM2_P
P87	F	6 y	Pre-lingual	Mild	No	AR	Deafness, autosomal recessive 1A	*GJB2* (NM_004004.6)	c.439G>A (p.Glu147Lys)	P	PM3_VS + PP1+PP3_S
P88	M	31 y	Post-lingual	Moderate	Yes	AD	Deafness, autosomal dominant 10	*EYA4* (NM_004100.5)	c.725G>T (p.Gly242Val)	VUS	PM2_P + PP3
P89	M	11 y	Pre-lingual	Profound	No	AR	Van Maldergem syndrome 1	*DCHS1* (NM_003737.4)	c.826C>T (p.Gln276Ter)	VUS	PM2_P + PP3
P90	M	5 y	Pre-lingual	Severe	No	AD; AR	Deafness, autosomal dominant 73; Deafness, autosomal recessive 84A	*PTPRQ* (NM_001145026.2)	c.6070G>A (p.Ala2024Thr)/c.6070G>A (p.Ala2024Thr)	VUS/VUS	PM2_P
P91	M	10 y	Pre-lingual	Severe	No	AD	Lissencephaly 9 with complex brainstem malformation	*MACF1* (NM_012090.5)	c.2953del (p.Glu985SerfsTer6)	VUS	PM2_P + PP3
P92	M	19 y	Post-lingual	Moderate	Yes	AD	Auditory neuropathy, autosomal dominant 1	*DIAPH3* (NM_001042517.2)	c.1124C>T (p.Pro375Leu)/c.180 + 5G>T	VUS/VUS	PM2_P + PP3/PM2_P + PP3
P93	F	6 y	Pre-lingual	Profound	No	AR	Deafness, autosomal recessive 28	*TRIOBP* (NM_001039141.3)	c.5185–2A>G	LP	PVS1+PM2_P
P94	F	7 y	Pre-lingual	Severe	Yes	AR	Deafness, autosomal recessive 23/Usher syndrome, type 1F	*PCDH15* (NM_001142769.3非MANE转录本)	c.4938_4941dup (p.Glu1648IlefsTer28)	VUS	PVS1_M + PM2_P
P95	F	34 y	Post-lingual	Moderate	Yes	AD	Deafness, congenital, with onychodystrophy, autosomal dominant	*ATP6V1B2* (NM_001693.4)	c.1414A>C (p.Thr472Pro)	VUS	PM2_P + PP3
P96	M	4 y	Pre-lingual	Severe	No	AR	Deafness, autosomal recessive 9	*OTOF* (NM_194248.3)	c.5098G>C (p.Glu1700Gln)	P	PM3_VS + PP1_S + PP3+PP4
P97	M	27 y	Pre-lingual	Severe	No	AR	Deafness, autosomal recessive 9	*OTOF* (NM_194248.3)	c.5816G>A (p.Arg1939Gln)	LP	PM2_P + PM3_S + PM5+PP3

Moreover, diagnostic proband-only ES revealed the presence of a single pathogenic or likely pathogenic variant in AR inheritance-pattern genes in 10 patients (10.8%). For example, patient 85 harbored the c.1251 + 5G>A variant in the *CEP78* gene. Studies have shown that this variant results in skipping of exon 10 and introduces a premature termination codon (Fu et al., 2017). It has also been reported in individual(s) with CEP78-related conditions (Fu et al., 2017). However, in the absence of another causal variant, the case remains inconclusive. Patient 87 harbored the c.439G>A (p.Glu147Lys) variant in the *GJB2* gene. In silico analysis supports that this missense variant has a deleterious effect on protein structure/function. This variant has been reported in multiple cases with hearing loss (Cryns et al., 2004; Frei et al., 2004), including homozygotes and individuals who were compound heterozygous for a second pathogenic variant. However, in the absence of a second pathogenic variant, the case remains inconclusive. Patient 96 harbored the c.5098G>C (p.Glu1700Gln) variant in the *OTOF* gene. This variant has been detected in at least 18 individuals with autosomal recessive non-syndromic hearing loss, including homozygotes and individuals who were compound heterozygous for a second pathogenic variant (Chiu et al., 2010; Zhu et al., 2021). However, in the absence of a second pathogenic variant, our case remains inconclusive. Patient 97 harbored the c.5816G>A (p.Arg1939Gln) variant in the *OTOF* gene. This variant is present in population databases (gnomAD 0.07%). This missense change has been observed in individuals with auditory neuropathy spectrum disorder and/or deafness (Bae et al., 2013; Iwasa et al., 2022). In at least one individual, the data are consistent with being in trans from a pathogenic variant. The variant was predicted as probably damaging by multiple computational algorithms in VarSome. However, in the absence of another causal variant, the case remains inconclusive.

## 4 Discussion

**FIGURE 2:**
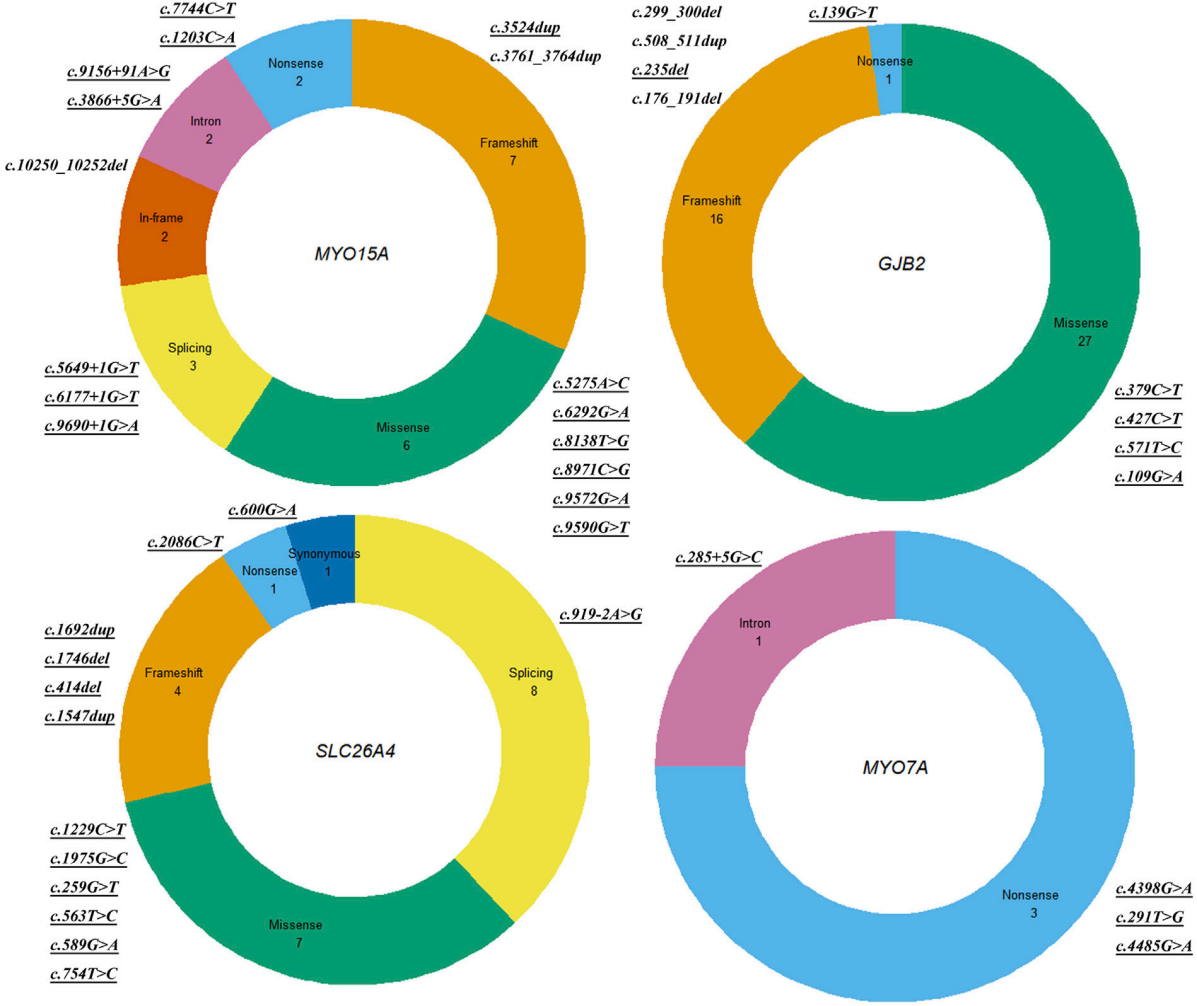
Detailed distribution of variants in *MYO15A*, *GJB2*, *SLC26A4*, and *MYO7A* genes. Note: Sector areas represent the frequencies or proportions of specific variant types detected in the gene, illustrating their relative distribution among the genetic alterations.

HL is one of the most etiologically heterogeneous disorders, encompassing over 400 genetic syndromes that include HL as a feature, more than 120 genes associated with NSHL, and a variety of non-genetic causes (Alford et al., 2014). With the advent of NGS technologies, it is now feasible to analyze hundreds of candidate HL genes simultaneously in a cost-effective manner. Nevertheless, the diagnostic yield varies across different patient cohorts and depends on the detection methods employed. Factors such as the degree of HL, the onset age of HL, the existence of family history, the ethnic origin, and the number of genes contained in the NGS panel may impact the rate of genetic diagnosis (García-García et al., 2020). Typically, the detection rate obtained usually increases in patients with a positive family history of HL or in cases where the HL was congenital and symmetric (Zhu et al., 2021). In the present study, ES was used to investigate the molecular etiology of HL in a Chinese cohort, resulting in genetic diagnoses for 78 of 171 probands, with an overall diagnostic yield of 45.6%. The diagnostic yield is comparable to that observed in other NGS-based panels on unselected heterogeneous HL patients, which ranged from 39% to 48% (Liu et al., 2022; Sloan-Heggen et al., 2016; Baux et al., 2017; Shearer et al., 2013). The study cohort consisted of patients with various types of sensorineural/mixed HL, including congenital, pre-lingual, and post-lingual HL, along with varying degrees of severity (mild, moderate, severe, and profound), and both stable and progressive HL and non-syndromic or syndromic HL. The heterogeneity within the patient cohort may affect the rate of genetic diagnosis. The positive diagnostic rate was higher in probands with a family history of HL (59.2%, 16/27), severe or profound HL (55.2%, 74/134), stable HL (54.4%, 68/125), symmetric HL (51%, 73/124), and pre-lingual HL (49.6%, 72/145), and it was lower in probands with mild or moderate HL (11.1%, 4/36), aminoglycoside exposure (19.0%, 4/21), a passed newborn hearing screening (20.0%, 6/30), post-lingual HL (23.1%, 6/26), and fluctuating HL (25.0%, 10/40). Moreover, a portion of the HL patients at our hospital opted for a stepwise approach and were pre-excluded for the *GJB2*, *SLC26A4*, and *MT-RNR1* gene variations prior to ES. It also explains why the proportions of *GJB2* and *SLC26A4* gene variations are comparatively low in the study cohort (Figures 1, 2). In addition, WES and CES were used as diagnostic tools for HL in the present study, and their efficacy was evaluated. The diagnostic yields were 44.1% (52 out of 118 probands) for WES and 49.0% (26 out of 53 probands) for CES. The raw data volume was approximately 9.7 GB for each sample tested by WES and approximately 5.1 GB for each sample tested by CES. The average coverage depth was 148.3 X and 263.4 X for WES and CES, respectively, with >99.5% of the target regions covered by at least 20 reads. The cost was 824 USD and 668 USD for WES and CES, respectively. The turn-around time for both assays was similar, which was 3–4 weeks.

In the present study, several novel variants were detected, and their pathogenicity was estimated according to the ACMG guidelines. As shown in Table 4, 28 novel variants from 21 genes were detected. Among the 28 novel variants identified, there were 13 missense variants, five frameshift variants, five nonsense variants, three intronic variants affecting splicing, and two canonical splicing variants. Six variants predicted to generate direct stop codons and one variant predicted to affect splicing were classified as pathogenic or likely pathogenic, and the remaining 21 variants were classified as VUS. The detection and evaluation of novel variants are important for expanding the spectrum of variants associated with HL.

**TABLE 4:** Classification of the novel variants identified in this study.

Gene	Nucleotide (protein)	Classification	Frequency		Pathogenicity scores		
			GnomAD exomes	GnomAD genomes	Revel	Conservation score (GERP)	SpliceAI
*ADGRV1*(NM_032119.4)	c.18374A>C (p.His6125Pro)	VUS = PM2_P	0	0	0.594	5.85	—
*ADGRV1*(NM_032119.4)	c.10119T>G (p.Tyr3373Ter)	LP = PVS1+PM2_P	0	0	NA	5	DS_DL = 0.18
*ATP6V1B2*(NM_001693.4)	c.1414A>C (p.Thr472Pro)	VUS = PM2_P + PP3+PP4	0	0	0.684	5.32	—
*CDH23*(NM_022124.6)	c.7661-1G>C	LP = PVS1+PM2_P	0	0	NA	5.02	DS_AL = 0.988
*DCHS1*(NM_003737.4)	c.826C>T (p.Gln276Ter)	VUS = PM2_P + PVS1_S	0	0	NA	5.01	—
*DHX16*(NM_003587.5)	c.2474C>T (p.Ser825Phe)	VUS = P2S_M + PM2_P + PP2+PP4	0	0	0.577	4.89	—
*DIAPH3*(NM_001042517.2)	c.1124C>T (p.Pro375Leu)	VUS = PM2_P + PP3+PP4	0.000065	0.000064	0.7	4.63	—
*DIAPH3*(NM_001042517.2)	c.180 + 5G>T	VUS = PM2_P + PP3+PP4	0	0	NA	5.25	DS_DL = 0.87
*ESRRB*(NM_001379180.1)	c.578-1G>C	VUS = PVS1_S + PM2_P	0	0	NA	4.92	DS_AL = 0.995
*ESRRB*(NM_001379180.1)	c.809G>A (p.Arg270Gln)	VUS = PM2_P + PP3_M	3	0	0.847	4.56	—
*EYA4*(NM_004100.5)	c.725G>T (p.Gly242Val)	VUS = PM2_P + PP3+PP4	0	0	0.625	5.99	DS_AL = 0.20
*FOXI1*(NM_012188.5)	c.575–522C>G	VUS = PM2_P	0	2	NA	3.25	DS_DL = 0.02
*MACF1*(NM_012090.5)	c.2953delG (p.Glu985SerfsTer6)	VUS = PM2_P + PVS1_M	0	0	NA	5.21	—
*MCM2*(NM_004526.4)	c.993dup (p.Cys332ValfsTer23)	VUS = PM2_P + PP3	0	0	NA	5	—
*MYO15A* (NM_016239.4)	c.3761_3764dup (p.Gly1256HisfsTer60)	P=PVS1+PM2_P + PM3_P	0	0	NA	4.76	—
*MYO15A* (NM_016239.4)	c.9590G>T (p.Ser3197Ile)	VUS = PM2_P	5	0	0.486	4.94	—
*MYO15A* (NM_016239.4)	c.9156 + 91A>G	VUS = PM2_P + PP3	0	0	NA	3.7	DS_DG = 0.302
*MYO15A* (NM_016239.4)	c.7744C>T (p.Gln2582Ter)	LP = PVS1+PM2_P	0	0	NA	5.25	DS_DG = 0.82
*MYO15A* (NM_016239.4)	c.8971C>G (p.Pro2991Ala)	VUS = PM2_P + PM3	2	0	0.113	4.71	—
*OTOA* (NM_144672.4)	c.119C>G (p.Ala40Gly)	VUS = PM2_P	0	0	0.178	6.02	DS_DL = 0.10
*PCDH15*(NM_001142769.3)	c.4938_4941dup (p.Glu1648IlefsTer28)	VUS = PM2_P + PVS1_M	0	0	NA	5.49	—
*PDE1C*(NM_001191057.4)	c.1174A>C (p.Met392Leu)	VUS = PM2_P	0	0	0.331	5.11	—
*PLS1*(NM_001145319.2)	c.1775G>A (p.Arg592Gln)	VUS = PM2_P + PP3_M	1	0	0.894	5.7	—
*POU4F3*(NM_002700.3)	c.952del (p.Val318Ter)	LP = PVS1_S + PM2_P + PP4	0	0	NA	4.62	—
*SOX10*(NM_006941.4)	c.580del (p.Glu194ArgfsTer92)	LP = PVS1+PM2_P	0	0	NA	4.24	—
*SOX2*(NM_003106.4)	c.137A>G (p.Asn46Ser)	VUS = PM2_P	0	0	0.616	2.29	—
*TMC1*(NM_138691.3)	c.1547G>A (p.Trp516Ter)	P=PVS1+PM2_P + PM3_P + PP4	1	0	NA	5.8	—
*TMPRSS3*(NM_001256317.3)	c.1075G>A (p.Ala359Thr)	VUS = PM2_P + PM3	1	0	0.511	4.94	—

Moreover, NGS panels enable the simultaneous analysis of hundreds of genes, and, in some cases, pathogenic variants in different genes can be identified in a single patient (García-García et al., 2020). In the study cohort, five cases harbored pathogenic variants in different genes or multiple pathogenic variants in the same gene. Patient 18 carried the c.676C>T (p.Arg226Ter) and c.880C>T (p.Arg294Trp) variants in the *PEX13* gene in the compound heterozygous state, in addition to the homozygous c.109G>A (p.Val37Ile) variant in the *GJB2* gene. Patient 23 carried the heterozygous c.119C>G (p.Ala40Gly) variant in the *OTOA* gene and 16p12.2 (chr16:21415697-21747711)×1 (including OTOA exon1-22), in addition to the homozygous c.109G>A (p.Val37Ile) variant in the *GJB2* gene. Patient 49 harbored c.18374A>C (p.His6125Pro) and c.10119T>G (p.Tyr3373Ter) in the *ADGRV1* gene in the compound heterozygous state, in addition to c.9690 + 1G>A and c.9590G>T (p.Ser3197Ile) in the *MYO15A* gene in the compound heterozygous state. Patient 39 harbored c.7744C>T (p.Gln2582Ter) and c.8971C>G (p.Pro2991Ala) in the *MYO15A* gene in the compound heterozygous state, in addition to c.993dup (p.Cys332ValfsTer23) in an AD-inherited *MCM2* gene variant. The patient from case 4,377, who presented with HL and EVA, was found to carry three heterozygous LP variants in the *SLC26A4* gene, namely, c.1547dupC (p.Ser517PhefsTer10), inherited from her father, and c.563T>C (p.Ile188Thr) and c.1746delG (p.Ala584ArgfsTer2), both inherited from her hearing mother. We speculate that c.1547dupC may contribute to the HL in this case, but we cannot determine whether c.563T>C and c.1746delG contribute respectively or collaboratively to the HL. These findings have important implications for reproductive genetic counseling, including risk assessment for the affected offspring and the potential application of pre-natal or pre-implantation genetic diagnosis.

Furthermore, the rapid accrual of knowledge in genomic medicine has prompted the reanalysis of existing data (Liu et al., 2019). As new disease genes are published, variants may be reclassified, and expanded phenotypic information becomes available (Ewans et al., 2018). This, combined with enhancements to bioinformatics pipelines and filtering strategies, contributes to an increased diagnostic yield, reinforcing the need for reanalysis (Ewans et al., 2018). In the present cohort, 93 probands (54.4%) remained undiagnosed after reanalysis and follow-up studies. One possible explanation for this is the clinical and genetic heterogeneity of HL. Additionally, causal variants may remain unidentified due to limitations in the analytical methods or inadequate knowledge in the literature regarding the genetics of the disease. Several factors may contribute to the improved diagnostic yield, including the strengthening of gene–disease associations, updates in the literature, the addition of detailed patient phenotype information, further sequencing of trios and other affected family members, improvements in sequencing data through re-sequencing, advancements in bioinformatics tools, and increased research collaborations facilitated by international case-sharing platforms (Shearer et al., 2013). These factors highlight the importance of reanalysis as the discovery of new gene– and variant–disease associations, the emergence of novel patient phenotypes, and the continued advancement of sequencing and bioinformatics technologies may collectively enhance the diagnostic yield of exome sequencing over time (Fung et al., 2020).

Nevertheless, ES has inherent technical constraints, including the inability to detect deep intronic variants, limited coverage of regulatory regions, and difficulty in identifying repeat expansions and complex structural variants. To overcome these limitations, multi-platform approaches should be integrated to enhance the diagnostic yield. Whole-genome sequencing (WGS) provides uniform coverage of coding and non-coding regions, enabling the detection of structural variants and regulatory mutations. Long-read sequencing resolves repetitive regions and phasing challenges. WGS-first pipelines are becoming clinically viable, although ES remains cost-effective for Mendelian disorders with clear phenotype–genotype correlations (Kang et al., 2025). Combining transcriptomics (RNA-seq) can reveal splicing impacts of non-coding variants, while machine learning models can improve non-coding variant interpretation.

## 5 Conclusion

Exome sequencing facilitates genetic diagnosis and improves the management of patients with HL in clinical practice. Identifying the etiology of HL may improve the management of patients with HL, refine genetic counseling, and facilitate the estimation of recurrence risk (Alford et al., 2014). The study highlights the importance of regularly reevaluating non-diagnostic exomes in light of updated gene discoveries, expanding variant databases, and improving bioinformatics pipelines to maximize diagnostic yield.

## Data Availability

The datasets generated and/or analyzed during the current study are not publicly available due to individual privacy but are available from the corresponding author (Aihua Yin, E-mail: yinaihua0131@163.com) on reasonable request.

## Abbreviations

HL, hearing loss; SHL, syndromic hearing loss; NSHL, non-syndromic hearing loss; NGS, next-generation sequencing; WGS, whole-genome sequencing; WES, whole-exome sequencing; CES, clinical exome sequencing; ES, exome sequencing; AR, autosomal recessive; AD, autosomal dominant; ACMG, American College of Medical Genetics and Genomics; AMP, Association for Molecular Pathology; P, pathogenic; LP, likely pathogenic; VUS, variants of uncertain clinical significance; CNV, copy number variation; MAF, minor allele frequency.
